# Elucidating a Complex Mechanism

**DOI:** 10.1093/function/zqad051

**Published:** 2023-09-29

**Authors:** Victor Wray

**Affiliations:** Department of Structural Biology, Helmholtz Centre for Infection Research, Inhoffenstrasse 7, D-38124 Braunschweig, Germany

**Keywords:** ATP hydrolysis, F_1_-ATPase, Boyer’s binding change mechanism, Nath’s torsional mechanism, bi-site versus tri-site models of ATP synthesis/hydrolysis, ligand displacement

## A Perspective on “Beyond Binding Change: The Molecular Mechanism of ATP Hydrolysis by F_1_-ATPase and Its Biochemical Consequences”

Our understanding of the complex dynamic system driven by conformational change during adenosine triphosphate (ATP) hydrolysis by F_1_-ATPase is of fundamental biochemical importance.^1^, ^2^ Cryo-electron microscopy (Cryo-EM) studies^3^^‒^^5^ have contributed valuable structural information on how the F_1_-ATPase functions, although, in themselves, these have not led to a definitive mechanism. The F_1_-ATPase is a multi-subunit system containing 3 β-catalytic sites that have been studied by biophysical single-molecule experiments based on direct visualization of the rotation of its central γ-subunit.^6^ However, it is difficult to establish which interconverting site or sites contribute energy for the observed rotation, given that a site can perform the elementary chemical steps of ATP binding, ATP hydrolytic bond cleavage, and product (Pi and adenosine diphosphate, ADP) release.^7^

Originally, the molecular mechanism of ATP synthesis/hydrolysis was studied using classical biochemical approaches that provided a wealth of fundamental data. A bi-site Boyer’s binding change mechanism of ATP synthesis/hydrolysis (Nobel Prize for Chemistry, 1997) was postulated between 1973 and 1993 based on biochemical unisite/multisite catalysis and oxygen exchange experiments.^8^ An alternative tri-site Nath’s torsional mechanism of energy transduction and ATP synthesis/hydrolysis was first proposed in 1999 and developed over the next 25 yr using a novel multidisciplinary approach,^9^ which integrated physics, chemistry, biochemistry, and engineering. The direct measurements by Senior and coworkers of the fluorescence quenching of tryptophan probes inserted into the β-catalytic sites of F_1_-ATPase established that ATP hydrolysis takes place by a tri-site mechanism.^1^ A result that has gained universal acceptance in the field and contradicts a fundamental tenet of Boyer’s binding change mechanism.^8^

In a recent publication in *Frontiers in Chemistry*, various key puzzling aspects of the hydrolysis mechanism have been identified and a new rationale is proposed.^10^ It is shown that previously postulated models, including those based on structural and single-molecule microscopy data, involve effectively bi-site mechanisms as catalysis and γ rotation occur with only 2 of the 3 β-catalytic sites occupied by nucleotides. Thus, the models contradict the experimental data of Senior and colleagues^1^ and therefore require modification. Senior and coworkers did propose a true tri-site model of catalysis by the F_1_-ATPase; however, their model has been shown to contradict^10^ longstanding facts from the single-molecule studies.^6^

**Figure 1.**
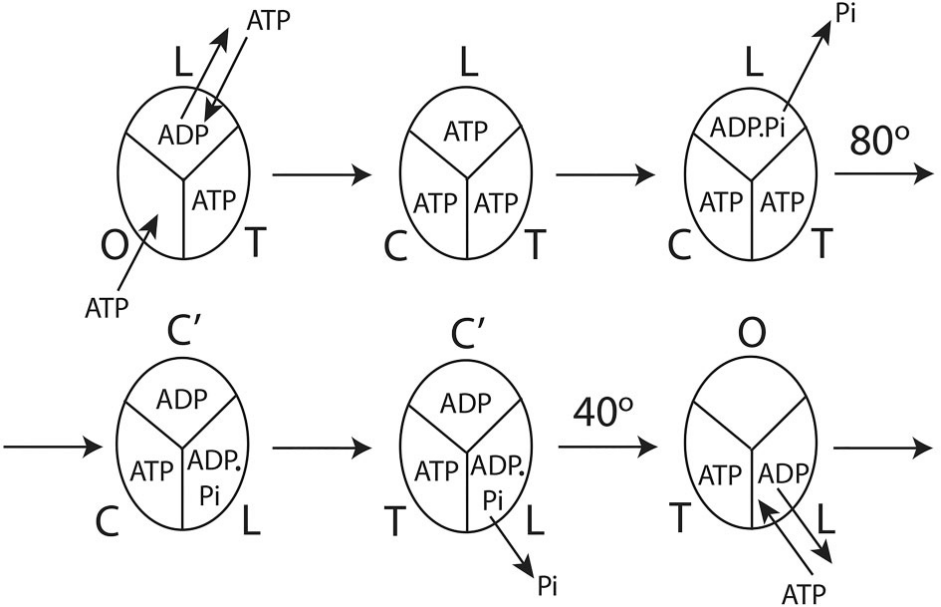
New molecular mechanism of steady-state *V*_max_ hydrolysis by F_1_-ATPase based on Nath’s torsional mechanism of energy transduction and ATP synthesis/hydrolysis.^10^ Boyer's binding change mechanism conceives that release of the product ADP results from binding of ATP to an allosteric site.^8^ On the basis of biochemical cold chase experiments, the new mechanism postulates activation by the simpler, more general explanation involving ligand displacement at a catalytic site of the F_1_-ATPase (panels 1 and 6 in Figure 1). For details, consult Nath (2023)^10^.

In the *Frontiers* publication, Nath offers a solution to these elusive problems by further in-depth considerations based on new biochemical unisite/cold chase experiments.^10^ The concept of ligand displacement, ie, exchange of bound ADP by medium ATP had already been a part of his mechanism.^7^ Now, it is shown that the ATP bound in site 2, after exchange of the ADP, also undergoes ATP hydrolytic bond cleavage and releases Pi, and these latter chemical events are postulated to drive the sub-step of γ-subunit rotation from 0° to 80°/90°.^10^ This makes the proposed model^10^ a true tri-site mechanism of steady-state hydrolysis that essentially completes the mechano-chemical coupling scheme (Figure 1). Since the movement of Pi from bound ADP is quantized in sub-steps, the pause/dwell observed in single-molecule experiments on human and mitochondrial^6^ F_1_-ATPase at an intermediate angle is also explained. Unisite catalysis has been known for decades, however, due to exclusive focus on the characteristics of the high-affinity catalytic site, the binding and subsequent hydrolysis of promoter ATP at a second site were not detected, as explained,^10^ and also revealed by a recent cryo-EM study.^3^ Acknowledging the biochemical basis of unisite catalysis by F_1_-ATPase on loading sub-stoichiometric ATP in site 1 and the biochemical framework of the cold chase experiments, one should accept the above biochemical results on the fate of bound ATP in site 2. The novel steady-state model of *V*_max_ synthesis/hydrolysis by F_1_-ATPase^10^ explains the requirement for three catalytic sites. It also clarifies the irreversibility of the two modes of catalysis, and explains why hydrolysis is not a simple reversal of ATP synthesis. These key mechanistic aspects arise from the fact that medium ADP is unable to displace bound ATP from a catalytic site.^10^

The proposed molecular mechanism indicates that the macroscopic reversal of the synthase and hydrolase activities is not merely a chemical reaction operating in reverse. Yet, the F_1_F_o_-ATP synthase mechanism,^2^ paradoxically proposed almost a decade before the ATP hydrolysis mechanism,^7^ is now seen to be the microscopic reverse of the complex mechanism of ATP hydrolysis.^10^ Nath’s torsional mechanism of ATP synthesis/hydrolysis shows that conformational changes at the bottom and top of the central stalk of ATP synthase are essential requirements.^2, 7, 10^ These are absent in other rotary mechanisms [see Nath (2023)^10^] that have attempted to describe the working of this complex molecular machine.

Fortunately, sufficient journal space has been allotted to show how the new detailed mechanism of ATP hydrolysis^10^ helps interpret previously determined X-ray and cryo-EM structures of F_1_-ATPase. These can now be considered as structural snapshots within the steady-state cycle and allow recognition of the different angular positions of the central γ-subunit and the ε-subunit arising from changes in the conformational states of the β-catalytic sites, as well as affording a wealth of other novel mechanistic insights.^10^ For example, Nath’s analysis shows how the hydrolysis mechanism of the α_3_β_3_γ subcomplex, or with truncated ε-subunit,^3^, ^4^ differs from that with fully constituted F_1_.^10^ The mechanism is in agreement with the conclusions from cryo-EM studies that “F_o_F_1_ and V/A-ATPase share a common tri-site mechanism.”^4^ However, defining an ATPase mechanism as tri-site because nucleotide occupancy transitions between 2 and 3 binding sites during continuous catalysis [see Nakano et al. (2023)^4^] is inadequate. For a mechanism to be truly tri-site, “catalysis must occur, and rotation must take place during steady-state *V*_max_ hydrolysis only when all three catalytic sites are occupied by bound Mg-nucleotide.”^10^

Disquietingly, the new structural^3^^‒^^5^ and single-molecule studies^6^ fail to mention the original tri-site Nath’s torsional mechanism that highlighted the role of torsion in the central stalk and the regulation of synthase mechanism by the ε-subunit [see Nath (2002, 2008, 2023)^2, 7, 10^ and references therein]. In future, a balanced appreciation of the current status of research can only be afforded by the insistence of referencing all relevant work. A straightforward solution is to ensure that papers that cite the binding change mechanism also contain references to the torsional mechanism.

A large body of work has now shown that the ATP synthase is a molecular constituent of the permeability transition pore in mitochondria. Hence, a detailed knowledge of ATP synthesis and hydrolysis mechanisms can help researchers establish a molecular basis of apoptosis and cell death, with manifold applications to disease. Improved future technological innovations need to include the development of hybrid structural techniques at atomic resolution that can simultaneously monitor rotation/conformational changes and the catalytic state of the binding sites with their nucleotide occupancies. These will include improvements in the resolution of time-resolved spectroscopic techniques for probing enzyme dynamics as well as complementary computer simulations. In the long-term, the current debate over the use of artificial intelligence and machine learning will hopefully be directed toward its development for verification of theoretical models of biochemical phenomena. In any case, the work by Nath (2023)^10^ is a major breakthrough for our understanding of the molecular mechanism of ATP hydrolysis by F_1_-ATPase.
